# A dataset describing data discovery and reuse practices in research

**DOI:** 10.1038/s41597-020-0569-5

**Published:** 2020-07-13

**Authors:** Kathleen Gregory

**Affiliations:** Data Archiving and Networked Services, Royal Netherlands Academy of Arts & Sciences, Anna van Saksenlaan 51, 2593 HW Den Haag, The Netherlands

**Keywords:** Research data, Publishing, Social sciences

## Abstract

This paper presents a dataset produced from the largest known survey examining how researchers and support professionals discover, make sense of and reuse secondary research data. 1677 respondents in 105 countries representing a variety of disciplinary domains, professional roles and stages in their academic careers completed the survey. The results represent the data needs, sources and strategies used to locate data, and the criteria employed in data evaluation of these respondents. The data detailed in this paper have the potential to be reused to inform the development of data discovery systems, data repositories, training activities and policies for a variety of general and specific user communities.

## Abstract (summary)

**Table:** 

Measurement(s)	data discovery behaviours • data reuse behaviours • data management
Technology Type(s)	Survey • Self-Report

Machine-accessible metadata file describing the reported data: 10.6084/m9.figshare.12445034

## Background & Summary

Reusing data created by others, so-called secondary data^1^, holds great promise in research^2^. This is reflected in the creation of policies^3^, platforms (i.e. the European Open Science Cloud^4^), metadata schemas (i.e. the DataCite schema^5^) and search tools, i.e. Google Dataset (https://datasetsearch.research.google.com/) or DataSearch (https://datasearch.elsevier.com), to facilitate the discovery and reuse of data. Despite the emergence of these systems and tools, not much is known about how users interact with data in search scenarios^6^ or the particulars of how such data are used in research^7^.

This paper describes a dataset first analysed in the article *Lost or Found? Discovering data needed for research*^8^. The dataset includes quantitative and qualitative responses from a global survey, with 1677 complete responses, designed to learn more about data needs, data discovery behaviours, and criteria and strategies important in evaluating data for reuse. This survey was conducted as part of a project investigating contextual data search undertaken by two universities, a data archive, and an academic publisher, Elsevier. The involvement of Elsevier enabled the recruitment strategy, namely drawing the survey sample from academic authors who have published an article in the past three years that is indexed in the Scopus literature database (https://www.scopus.com/).This recruitment strategy helped to ensure that the sample consisted of individuals active in research, across disciplines and geographic locations.

The data themselves are presented in two data files, according to the professional role of respondents. The dataset as a whole consists of these two data files, one for researchers (with 165 variables) and one for research support professionals (with 167 variables), the survey questionnaire and detailed descriptions of the data variables^9^. The survey questionnaire contains universal questions which could be applicable to similar studies; publishing the questionnaire along with the data files not only facilitates understanding the data, but it also fosters possible harmonization with other survey-based studies.

The dataset has the potential to answer future research questions, some of which are outlined in the usage notes of this paper, and to be applied at a practical level. Designers of both general and specific data repositories and data discovery systems could use this dataset as a starting point to develop and enhance search and sensemaking interfaces. Data metrics could be informed by information about evaluation criteria and data uses present in the dataset, and educators and research support professionals could build on the dataset to design training activities.

## Methods

The description below of the methods used to design the questionnaire and to collect the data, as well as the description of potential biases in the technical validation section, all build on those presented in the author’s previous work^8^.

### Questionnaire design

**Fig. 1:**
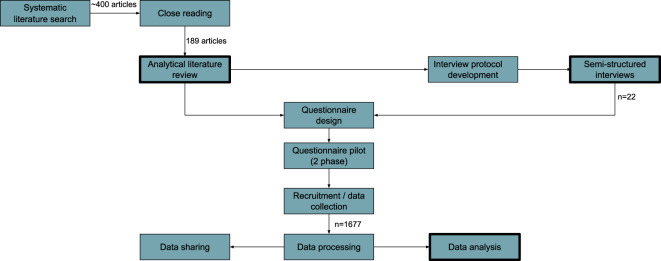
Creation of dataset in relation to prior empirical work by the author. Bolded rectangles indicate steps with associated publications, resulting from an analytical literature review^10^, semi-structured interviews^11^ and an analysis of the survey data^8^.

The author’s past empirical work investigating data search practices^10,11^ (see also Fig. 1), combined with established models of interactive information retrieval^12–15^ and information seeking^16,17^ and other studies of data practices^18,19^, were used to design questions examining the categories identified in Table 1. Specifically, questions explored respondents’ data needs, their data discovery practices, and their methods for evaluating and making sense of secondary data.

**Table 1:** Content and branches of questionnaire items listed according to questionnaire section.

Category	Content	Branches
**Demographics**	Role	If research support professional (RSP) - asked all questions plus those marked *RSP only*; not asked questions marked *R only* If not RSP - asked all questions marked R only; not asked questions marked *RSP only*
	Discipline*	
	Years professional experience	
	Country of employment	
	Organization type	
	Perceptions (own, others) about data sharing	
	Perceptions (own, others) about data reuse	
	Ever shared data (*R only)*	
**Data needs**	Describe needed data	
	Select data type needed*	
	Purpose for using secondary data*	
	Need data outside of discipline (*R only)*	If yes - how find these data *(R only)*
	Need data to support others/own research *(RSP only)*	If support others - Who do you support* *(RSP only)*
		- How do you provide support* *(RSP only)*
**Discovering**	Who finds data for you* *(R only)*	
	Sources used to find data - frequency of use	If academic literature - how use literature?* If search engine - how successful?
	Active vs serendipitous data discovery	
	Social interactions in discovery and access**	
	Discover literature differently than data	If not no - describe differences
	Ease of finding data *(R only)*	If not easy - what are the challenges?*
**Evaluating/sensemaking**	Social interactions in evaluating/sensemaking**	
	Important information about data when evaluating	
	Differences between self and those support *(RSP only)*	
	Evaluation/sensemaking strategies	
	Important aspects in establishing trust	
	Important aspects in establishing quality	

Questions only asked to research support professionals are marked with RSP; those only asked to other respondents are marked with R. Items that allowed multiple responses are marked with an asterisk. Items marked with a double asterisk correspond to the same multiple response question.

The questionnaire used a branching design, consisting of a maximum of 28 primarily multiple choice items (Table 1). The final question of the survey, which provided space for respondents to provide additional comments in an open text field is not represented in Table 1. The individual items were constructed in accordance with best practices in questionnaire design, with special attention given to conventions for wording questions and the construction of Likert scale questions^20,21^. Nine of the multiple choice questions were constructed to allow multiple responses. There were a maximum of three optional open response questions. The majority of multiple choice questions also included the possibility for participants to write-in an “other” response.

The first branch in the questionnaire design was based on respondents’ professional role. Respondents selecting “librarians, archivists or research/data support providers,” a group referred to here as *research support professionals*, answered a slightly different version of the questionnaire. The items in this version of the questionnaire were worded to reflect possible differences in roles, i.e. whether respondents seek data for their own use or to support other individuals. Four additional questions were asked to research support professionals in order to further probe their professional responsibilities; four questions were also removed from this version of the questionnaire. This was done in order to maintain a reasonable completion time for the survey and because the removed questions were deemed to be more pertinent to respondents with other professional roles, i.e. researchers. The questionnaire is available in its entirety with the rest of the dataset^12^.

### Sampling, recruitment and administration

Individuals involved in research, across disciplines, who seek and reuse secondary data comprised the population of interest. This is a challenging population to target, as it is difficult to trace instances of data reuse, particularly given the fact that data citation, and other forms of indexing, are still in their infancy^22^. The data reuse practices of individuals in certain disciplines have been better studied than others^23^, in part because of the existence of established data repositories within these disciplines^24^. In order to recruit individuals active in research across many disciplinary domains, a broad recruitment strategy was adopted.

Recruitment emails were sent to a random sample of 150,000 authors who are indexed in Elsevier’s Scopus database and who have published in the past three years. The recruitment sample was created to reflect the distribution of published authors by country within Scopus. Two batches of recruitment emails were sent: one of 100,000 and the other of 50,000. One reminder email was sent two weeks after the initial email. A member of the Elsevier Research and Academic Relations team created the sample and sent the recruitment letter, as access to the email addresses was not available to the investigator due to privacy regulations. The questionnaire was scripted and administered using the Confirmit software (https://www.confirmit.com/).

1637 complete responses were received during a four-week survey period between September and October 2018 using this methodology. Only seven of the 1637 responses came from research support professionals. In a second round of recruitment in October 2018, messages were posted to discussion lists in research data management and library science to further recruit support professionals. Individuals active in these lists spontaneously posted notices about the survey on their own Twitter feeds. These methods resulted in an additional 40 responses, yielding a total of 1677 complete responses.

### Ethical review and informed consent

This study was approved by the Ethical Review Committee Inner City faculties (ERCIC) at Maastricht University, Netherlands, on 17 May 2018 under the protocol number ERCIC_078_01_05_2018.

Prior to beginning the study, participants had the opportunity to review the informed consent form. They indicated their consent by clicking on the button to proceed to the first page of survey questions. Respondents were informed about the purpose of the study, its funding sources, the types of questions which would be asked, how the survey data would be managed and any foreseen risks of participation.

Specifically, respondents were shown the text below, which also states that the data would be made available in the DANS-EASY data repository (https://easy.dans.knaw.nl), which is further described in the Data Records section of this paper.

Your responses will be recorded anonymously, although the survey asks optional questions about demographic data which could potentially be used to identify respondents. The data will be pseudonymized (e.g. grouping participants within broad age groups rather than giving specific ages) in order to prevent identification of participants. The results from the survey may be compiled into presentations, reports and publications. The anonymized data will be made publicly available in the DANS-EASY data repository.

Respondents were also notified that participation was voluntary, and that withdrawal from the survey was possible at any time. They were further provided with the name and contact information of the primary investigator.

## Data Records

### Preparation of data files

The data were downloaded from the survey administration system as csv files by the employee from Elsevier and were sent to the author. The downloads were performed in two batches: the 1637 responses received before the additional recruiting of research support professionals, and the 40 responses received after this second stage of recruitment. The seven responses from research support professionals from the first round of recruitment were extracted and added to the csv file from the second batch. This produced separate files for research support professionals and the remainder of respondents, who are referred to as *researchers* in this description. This terminology is appropriate as the first recruitment strategy ensured that respondents were published academic authors, making it likely that they had been involved in conducting research at some point in the past three years.

The following formatting changes were made to the data files in order to enhance understandability for future data reusers. All changes were made using the analysis program R^25^.

- Open responses were checked for any personally identifiable information, particularly email addresses. This was done by searching for symbols and domains commonly used in email addresses (i.e. “@”; “.com,” and “.edu”). Two email addresses were identified in the final question recording additional comments about the survey. In consultation with an expert at the DANS-EASY data repository, all responses from this final question were removed from both data files as a precautionary measure.
- Variables representing questions asked only to research support professionals were removed from datadiscovery_researchers.csv. Variables representing questions asked only to researchers were removed from datadiscovery_supportprof.csv.
- Variables were renamed using mnemonic names to facilitate understanding and analysis. Variable names for questions asked to both research support professionals and researchers have the same name in both data files.
- Variables were re-ordered to match the order of the questions presented in the questionnaire. Demographic variables, including role, were grouped together at the end of the data files.
- Multiple choice options which were not chosen by respondents were recorded by the survey system as zeros. If a respondent was not asked a question, this is coded as “Not asked.” If a respondent wrote “NA” or a similar phrase in the open response questions, this was left unchanged to reflect the respondent’s engagement with the survey. If a respondent did not complete an optional open response question, this was recorded as a space, which appears as an empty cell. In the analysis program R, e.g., this empty space is represented as “ “.

### Description of data and documentation files

The dataset described here consists of one text readme file, four csv files, and one pdf file with the survey questionnaire. These files should be used in conjunction with each other in order to appropriately use the data. Table 2 provides a summary and description of the files included in the dataset.

**Table 2:** Description of files composing the dataset.

Data file name	Description	No. cases	No. variables
GREGORY_DATA_DISCOVERY_Readme.txt	Provides guidance to the dataset	*NA*	*NA*
datadiscovery_researchers.csv	Contains data for respondents identifying themselves as researchers, students, managers or other types of professionals	1630	165
datadiscovery_supportprof.csv	Contains data for respondents identifying themselves as librarians, archivists or research/data support providers	47	167
variable_labels_researchers.csv	Contains a description of the variable names in the datadiscovery_researchers.csv file	*NA*	*NA*
variable_labels_supportprof.csv	Contains a description of the variable names in the datadiscovery_supportprof.csv	*NA*	*NA*
datadiscovery_questionnaire.pdf	Contains the questionnaire for both researchers and research support professionals	*NA*	*NA*

Descriptions of the variable names are provided in two files (Table 2). Variables were named following a scheme that matches the structure of the questionnaire; each variable name begins with a mnemonic code representing the related research aim. The primary codes are summarised in Table 3. The values of the variables for multiple choice items are represented as either a “0” for non-selected options, as described above, or with a textual string representing the selected option.

**Table 3:** Description of primary mnemonic codes used to preface variable names.

Mnemonic code	Associated research aim
need_	Data needs
use_	Purposes for which data are used
find_	Data search and discovery practices
strategy_	Data discovery strategies Related to *find_*
source_	Sources used to discover data Related to *find_*
eval_	Data evaluation and sense-making
accss_	Data access
disc_	Discipline chosen
dem_	Demographic information

The dataset is available at the DANS-EASY data repository^9^. DANS-EASY is a principal component of the federated national data infrastructure of the Netherlands^26^ and is operated by the Data Archive and Networked Services (DANS), an institution of the Royal Netherlands Academy for Arts and Sciences and the Dutch Research Council. DANS-EASY has a strong history of providing secure long-term storage and access to data in the social sciences^27^. The repository has been awarded a CoreTrustSeal certification for data repositories (https://www.coretrustseal.org/), which assesses the trustworthiness of repositories according to sixteen requirements. These requirements focus on organisational infrastructure (e.g. licences, continuity of access and sustainability), digital object management (e.g. integrity, authenticity, preservation, and re-use) and technology (e.g. technical infrastructure and security).

### Sample characteristics

**Fig. 2:**
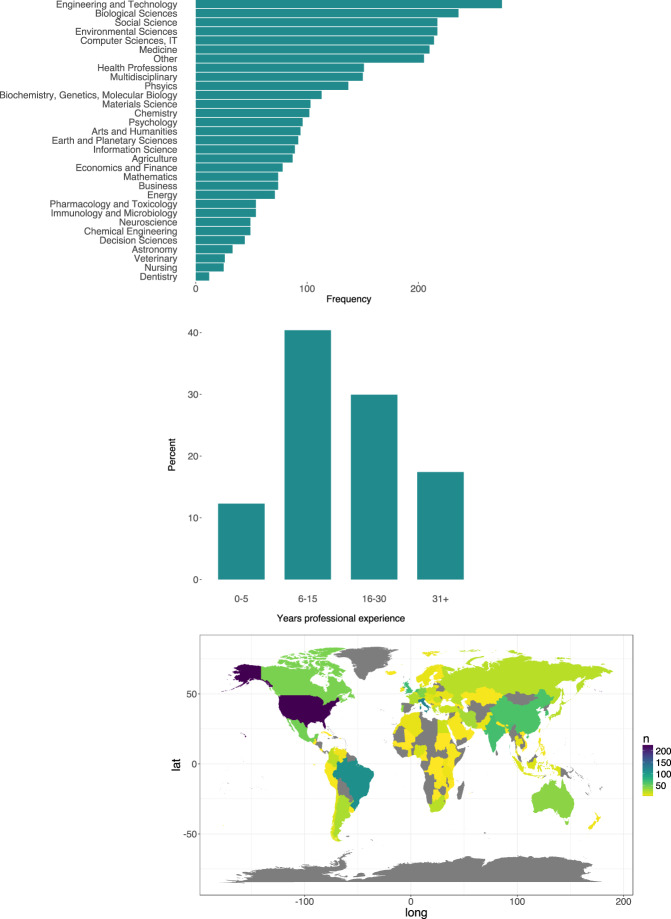
(**a**) Disciplinary domains selected by respondents; multiple responses possible (n = 3431). (**b**) Respondents’ years of professional experience; percentages denote percent of respondents (n = 1677). (**c**) Number of respondents by country of employment (n = 1677).

Respondents identified their disciplinary domains of specialization from a list of 31 possible domains developed after the list used by Berghmans, *et al*.^28^. Participants could select multiple responses for this question. The domain selected most often was engineering and technology, followed by the biological, environmental and social sciences (Fig. 2a) Approximately half of the respondents selected two or more domains, with one quarter selecting more than three.

Forty percent of respondents have been professionally active for 6–15 years (Fig. 2b). The majority identified as being researchers (82%) and are employed at universities (69%) or research institutions (17%). Respondents work in 105 countries; the most represented countries include the United States, Italy, Brazil and the United Kingdom (Fig. 2c).

## Technical Validation

Several measures were performed to ensure the validity of the data, both before and after data collection. Sources of uncertainty and potential bias in the data are also outlined below in order to facilitate understanding and data reuse.

### Questionnaire development

The questionnaire items were developed after extensively reviewing relevant literature^10,29–32^ and conducting semi-structured interviews to test the validity of our guiding constructs. To test the validity and usability of the questionnaire itself, a two-phase pilot study was conducted. In the first phase, four researchers, recruited using convenience sampling, were observed as they completed the online survey. During these observations, the researchers “thought out loud” as they completed the survey; they were encouraged to ask questions and to make remarks about the clarity of wording and the structure of the survey. Based on these comments, the wording of questions was fine tuned and additional options were added to two multiple choice items.

In the second pilot phase, an initial sample of 10,000 participants was recruited, using the primary recruitment methodology detailed in the methods section of this paper. After 102 participants interacted with the survey, the overall completion rate (41%) was measured and points where individuals stopped completing the survey were noted. Based on this information, option-intensive demographic questions (i.e. country of employment, discipline of specialization) were moved to the final section of the survey in order to minimize survey fatigue. The number of open-ended questions were also reduced and open-response questions were made optional.

The online presentation of the survey questions also helped to counter survey fatigue. Only one question was displayed at a time; the branching logic of the survey ensured that respondents were only shown the questions which were relevant to them, based on their previous answers.

### Questionnaire completion

1677 complete responses to the survey questionnaire were received. Using the total number of recruitment emails in the denominator, this yields a response rate of 1.1%. Taking into account the number of non-delivery reports which were received (29,913), the number of invalid emails which were reported (81) and the number of recruited participants who elected to opt-out of the survey (448) yields a slightly higher response rate of 1.4%. It is likely that not all of the 150,000 individuals who received recruitment emails match our targeted population of data seekers and reusers. Knowledge about the individuals who did not respond to the survey and about the frequency of data discovery and reuse within research as a whole, is limited; this complicates the calculation of a more accurate response rate, such as the methodology described in^33^.

A total of 2,306 individuals clicked on the survey link, but did not complete it, yielding a completion rate of 42%. Of the non-complete responses, fifty percent stopped responding after viewing the introduction page with the informed consent statement. This point of disengagement could be due to a variety of reasons, including a lack of interest in the content of the survey or a disagreement with the information in the consent form. The majority of individuals who did not complete the survey stopped responding within the first section of the survey (75% of non-complete responses). Only data from complete responses are included in this dataset.

Of the 1677 complete responses, there was a high level of engagement with the optional open response questions. Seventy-eight percent of all respondents answered Q2 regarding their data needs; 92% of respondents who were asked Q5a provided an answer; and 69% of respondents shown Q10a described how their processes for finding academic literature and data differ.

### Data quality and completeness

Checks for missing values and NAs were performed using standard checks in R. As detailed in the section on data preparation, multiple choice responses not selected by respondents were recorded as a zero. If a respondent was not asked a question, this was coded as “Not asked.” If a respondent wrote “NA” or a similar phrase in the open response questions, this was left unchanged to reflect the respondent’s engagement with the survey. If a respondent did not complete an optional open response question, this was recorded as a space, which appears as an empty cell. In the analysis program R, e.g., this empty space is represented as “ “.

Due to the limited available information about non-responders to the survey and about the frequency of data seeking and discovery behaviours across domains in general, the data as they stand are representative only of the behaviours of our nearly 1700 respondents - a group of data-aware people already active in data sharing and reuse and confident in their ability to respond to an English-language survey. Surveys in general tend to attract a more active, communicative part of the targeted population and do not cover non-users at all^34^. While not generalizable to broader populations, the data could be transferable^35,36^ to similar situations or communities. Creating subsets of the data, i.e. by discipline, may provide insights that can be applied to particular disciplinary communities.

There are potential sources of bias in the data. The recruited sample was drawn to mirror the distribution of published authors by country in Scopus; the geographic distribution of respondents does not match that of the recruited sample (Table 4). This is especially noticeable for Chinese participants, who comprised 15% of the recruited sample, but only 4% of respondents. This difference could be due to a number of factors, including language differences, perceived power differences^37^, or the possibility that data seeking is not a common practice.

**Table 4:** Percentage of recruited participants by geographic location compared to percentage of respondents providing complete responses.

Percent of recruited sample		Percent of respondents	
United States	19%	United States	13%
China	15%	Italy	7%
United Kingdom	5%	Brazil	7%
Germany	5%	United Kingdom	4%
Japan	4%	India	4%
France	4%	South Korea	4%
India	4%	Netherlands	4%
Italy	3%	China	4%
Canada	3%	Mexico	3%
Spain	3%	Canada	3%
Australia	3%	Germany	3%
South Korea	3%	Australia	2%
Brazil	2%	Portugal	2%
Netherlands	2%	Iran	2%
Russian Federation	1%	Argentina	2%
Taiwan	1%	France	2%
Iran	1%	Greece	1%
Swizerland	1%	Japan	1%
Turkey	1%	Russian Federation	1%
Poland	1%	South Africa	1%
Other	19%	Romania	1%

Our respondents were primarily drawn from the pool of published authors in the Scopus database. Some disciplinary domains are under-represented within Scopus, most notably the arts and humanities^38,39^. Subject indexing within Scopus occurs at the journal or source level. As of January 2020, 30.4% of titles in Scopus are from the health sciences; 15.4% from the life sciences; 28% from the physical sciences and 26.2% from the social sciences^45^. Scopus has an extensive and well-defined review process for journal inclusion; 10% of the approximately 25,000 sources indexed in Scopus are published by Elsevier^40^.

Self-reported responses also tend to be pro-attitudinal, influenced by a respondent’s desire to provide a socially acceptable answer. Survey responses can also be influenced by the question presentation, wording and multiple choice options provided. The pilot studies and the provision of write-in options for individual items helped to mitigate this source of error.

## Usage Notes

### Notes for data analysis

- It is key to note which questions were designed to allow for multiple responses. This will impact the type of analysis which can be performed and the interpretation of the data. These nine questions are marked with an asterisk in Table 1; the names of the variables related to these questions are summarized in Table 5.

**Table 5:** Content of questions which were designed to allow multiple responses and their associated variables.

Question content	Variable names
Discipline	all variables beginning with code *disc_*
Data type needed	need_obs, need_exp, need_sim, need_deriv, need_oth
Purpose for using secondary data	use_calb, use_bmk, use_vrf, use_inpt, use_idea, use_tch, use_nwprj, use_nwmth, use_tnd, use_cmp, use_smvs, use_intg, use_oth
Who do you support? (RSP)	whosupprt_stud, whosupprt_res, whosupprt_indus, whosupprt_oth
How do you support? (RSP)	supprt_teachdmp, supprt_teachsklls, supprt_finddta, supprt_curate, supprt_findlit, supprt_oth
Who finds data for you? (R)	find_whoself, find_whograd, find_whosuppt, find_whonetwk, find_whooth
How do you use academic literature?	strategy_goal, strategy_serend, strategy_cit, strategy_extrct, strategy_oth
What are challenges?	find_chalaccs, find_chalskill, find_chaldistrb, find_chaldigtl, find_chaltools, find_chalnetwk, find_chaloth
Social interactions in finding, accessing, evaluation/sensemaking	find_netwk, find_creatr, find_collab, find_conf, find_list, accss_netwk, accss_creatr, accss_collab, accss_conf, accss_list, eval_netwk, eval_creatr, eval_collab, eval_conf, eval_list

RSP indicates questions only asked to research support professionals. R indicates questions only present in the researcher data file.
- The data are available in standard csv formats and may be imported into a variety of analysis programs, including R and Python. The data are well-suited in their current form to be treated as factors or categories in these programs, with the exception of open response questions and the write-in responses to the “other” selection options, which should be treated as character strings. An example of the code needed to load the data into R and Python, as well as how to change the open and other response variables to character strings, is provided in the section on code availability. To further demonstrate potential analyses approaches, the code used to create Fig. 2a in R is also provided.
- Certain analysis programs, i.e. SPSS, may require that the data be represented numerically; responses in the data files are currently represented in textual strings. The survey questionnaire, which is available with the data files, contains numerical codes for each response which may be useful in assigning codes for these variables.
- Future users may wish to integrate the two data files to examine the data from all survey respondents together. This can easily be done by creating subsets of the variables of interest from each data file (i.e. by using the *subset* and *select* commands in R) and combining the data into a single data frame (i.e. using the *rbind* command in R). Variables that are common between both of the data files have the same name, facilitating this type of integration. An example of the code needed to do this is provided in the code for creating Fig. 2a.
- Open and write-in responses are included in the same data file with the quantitative data. These variables can be removed and analysed separately, if desired.
- To ease computational processing, the data do not include embedded information about the question number or the detailed meaning of each variable name. This information is found in the separate *variable_labels* csv file associated with each data file.

### Potential questions and applications

The data have the potential to answer many interesting questions, including those identified below.

1. How do the identified practices vary by demographic variables? The data could be sub-setted to examine practices along the lines of:

- Country of employment
- Career stage, e.g. early career researchers
- Disciplinary domain

(2) What correlations exist among the different variables, particularly the variables allowing for multiple responses? Such questions could examine Box 1–3:

- Possible correlations between the frequency of use of particular sources and the type of data needed or uses of data
- Possible correlations between particular challenges for data discovery and needed data or data use

(3) How representative are these data of the behaviours of broader populations?
(4) How will these behaviours change as new technologies are developed? The data could serve as a baseline for comparison for future studies.
(5) How do practices within a particular domain relate to the existence of data repositories and infrastructures within a domain? Given the practices identified in this survey, how can repositories and infrastructures better support data seekers and reusers?

Box 1. R code for loading data and changing selected columns to character strings. “‘{r}

*#Set the working directory; the data files should be in the working directory*.

setwd(“~/Desktop/survey/“)

*#Import the data files as data frames and store as “researcher.df” and “support.df”. If you don’t want to use factors, set stringsAsFactors = FALSE*.

researcher.df < - read.csv(file = ‘datadiscovery_researchers.csv’, header = TRUE, stringsAsFactors = TRUE)

support.df < - read.csv(file = ‘datadiscovery_supportprof.csv’, header = TRUE, stringsAsFactors = TRUE)

*#Select columns to be treated as character strings*.

cols.res < - c(“need_open”,“need_othresp”,“use_othresp”,“find_whoothresp”,“source_open”,“strategy_othresp”,“find_litdatopen”,“find_chalothresp”,“eval_infopen”,“eval_stratopen”,“eval_trstopen”,“eval_qualopen”,“disc_othresp”)

cols.sup < - c(“whosupprt_othresp”,“supprt_othresp”,“need_open”,“need_othresp”,“use_othresp”,“source_open”,“strategy_othresp”,“findaccseval_oth”,“find_litdatopen”,“eval_infopen”,“eval_spprtdopen”,“eval_stratopen”,“eval_trstopen”,“eval_qualopen”,“disc_othresp”)

*#Change these columns from factors to characters*

researcher.df[cols.res] < - lapply(researcher.df[cols.res], as.character)

support.df[cols.sup] < - lapply(support.df[cols.sup], as.character)

*#Check*

str(researcher.df)

str(support.df)

“‘

Box 2. Python code for loading data and changing selected columns to character strings. *#Import required libraries*

import pandas as pd

*#Read in the csv as a pandas dataframe. Pandas will infer data types but we will explicitly set all to “categories” initially and then change the “str” (string) columns later*. df = pd.read_csv(‘./datadiscovery_researchers.csv’, index_col = ‘responseid’, dtype = “category”)

*#Create a list of the columns which are not categories but should be treated as strings*.

str_cols = [‘need_open’,

       need_othresp’,

       use_othresp’,

       find_whoothresp’,

       source_open’,

       strategy_othresp’,

       find_litdatopn’,

       find_chalothresp’,

       eval_infopen’,

       eval_stratopen’,

       eval_trstopen’,

       eval_qualopen’,

       ‘disc_othresp’]

*#Change data type for columns to be treated as strings*.

df[str_cols] = df[str_cols].astype(“str”)

*#Print data types to confirm*

df[cat_cols].dtypes

df[str_cols].dtypes

Box 3. R code for creating Fig. 2a. “‘{r}

*#Install packages and libraries for plot*

install.packages(ggplot2)

install.packages(reshape2)

install.packages(dplyr)

library(ggplot2)

library(reshape2)

library(dplyr)

*#Select and combine variables from both data files to use in the plot*

researcherdisc.df < - subset(researcher.df, select = c(responseid,disc_agricul:disc_other))

supportdisc.df < - subset(support.df, select = c(responseid,disc_agricul:disc_other))

disc.df < - rbind(researcherdisc.df,supportdisc.df)

*#Transform data from wide to long*

disclong.df < - disc.df % > % melt(id.vars = c(“responseid”),value.name = “discipline”)

*#Create data frames with frequencies*

discfreq.df < - disclong.df % > %

       filter(discipline! = “0”) % > %

       select(responseid,discipline)% > %

       count(discipline)

*#Create plot of frequencies*

discplot < - ggplot(discfreq.df, aes(x = reorder(discipline, n), y = n)) + geom_bar(stat = “identity”, fill = “#238A8DFF”) + coord_flip()

*#Format plot and add labels*

discplot < - discplot + theme(plot.title = element_text(hjust = 0),axis.ticks.y = element_blank(),axis.text = element_text(size = 15),text = element_text(size = 15),panel.grid.major = element_blank(), panel.grid.minor = element_blank(),panel.background = element_blank()) + ylab(“Frequency”) + xlab(“Disciplinary domain”)

*#View plot*

discplot

“‘

## Data Availability

All R scripts used in data preparation and technical validation, along with the un-prepared data, are available upon request from the corresponding author. Examples of how to load the data and how to change factor/category columns to character columns in R (Box 1) and Python (Box 2) are provided. Additionally, the code used to create Fig. 2a in R (Box 3) is listed as an example of how to combine data from both data files into a single plot.
